# PI3K/AKT signaling drives titanium-induced angiogenic stimulus

**DOI:** 10.1007/s10856-020-06473-8

**Published:** 2021-01-27

**Authors:** Bruna Rodrigues Martins, Thais Silva Pinto, Célio Junior da Costa Fernandes, Fábio Bezerra, Willian Fernando Zambuzzi

**Affiliations:** Institute of Biosciences of Botucatu, Department of Chemical and Biological Sciences, UNESP – São Paulo State University, Botucatu, São Paulo Brazil

## Abstract

Although osseointegration and clinical success of titanium (Ti)-implanted materials depend on neovascularization in the reactional peri-implant tissue, very little has been achieved considering the Ti-molecules release on the behavior of endothelial cells. To address this issue, we challenged endothelial cells (HUVECs) with Ti-enriched medium obtained from two types of commercial titanium surfaces [presenting or not dual-acid etching (DAE)] up to 72 h to allow molecular machinery analysis. Our data show that the Ti-enriched medium provokes significant stimulus of angiogenesis-related machinery in endothelial cells by upexpressing VEGFR1, VEGFR2, VEGF, eNOS, and iNOS genes, while the PI3K/Akt signaling pathway was also significantly enhanced. As PI3K/AKT signaling was related to angiogenesis in response to vascular endothelial growth factor (VEGF), we addressed the importance of PI3K/Akt upon Ti-enriched medium responses by concomitantly treating the cells with wortmannin, a well-known PI3K inhibitor. Wortmannin suppressed the angiogenic factors, because VEGF, VEGFR1, and eNOS genes were downregulated in those cells, highlighting the importance of PI3K/AKT signaling on driving angiogenic phenotype and angiogenesis performance within the peri-implant tissue reaction. In conjunction, these data reinforce that titanium-implantable devices modify the metabolism of surrounding cells, such as endothelial cells, probably coupling osteogenesis and angiogenesis processes in peri-implant tissue and then contributing to successfully osseointegration of biomedical titanium-based devices.

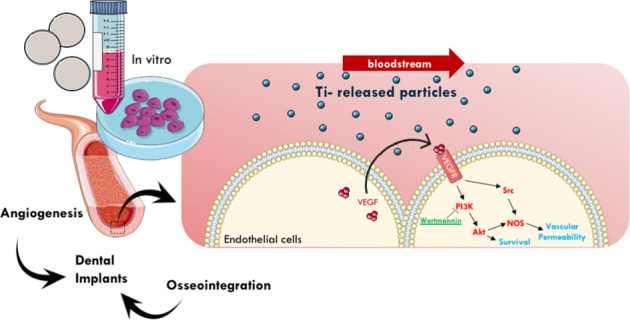

## Introduction

Titanium-based implants are widely used in dentistry and medical fields contributing to therapies for edentulism and stabilization of bone fractures, respectively, mainly due to their properties including mechanical strength, high corrosion resistance, low toxicity, and adequate biocompatibility acceptance [1–3]. However, current studies have shown that titanium alloys are not inert as once believed, and they dynamically release particles and molecules to their embedding microenvironment affecting the surrounding cell behavior, as osteoblast-like cells, and the vascular endothelium that irrigates the injured tissue and then affects the endothelial cells (ECs) [4, 5]. Because of that and especially considering vascularization, which is an important step to regenerate the tissue surrounding the implants, studies are necessary to address the effect of titanium on ECs.

For the biocompatibility and performance of biomaterials, some properties of the biomaterial surfaces should be consider critical issues that trigger signals of host tissue interactions [6]. As previously mentioned, adequate vascularization is required for successful tissue regeneration. Osseointegration is an important process that occurs in the appositional interface between tissue and biomaterial, in which an orchestrated action of bone formation and bone resorption helps to maintain the health of neoformed bone.

To improve the osseointegration, reducing patient recuperation time and decreasing implant-associated complications, several modifications on biomaterial surfaces have been widely proposed, such as nanotopographical and acid etching [7, 8]. These physicochemical properties require more investigation about their effects on biological performance for full comprehension of the mechanism underlying wound healing after an implant is introduced [1]. To better address this issue, our group has investigated the interaction between the biomaterial and the host tissue in a direct [9] and indirect way [10], presenting specific signaling cascades triggered by this interface. Although titanium has been considered for decades a resistant and inert material, it can release molecules triggering dynamic responses in its microenvironment and surrounding tissues [9], and it is reasonable to hypothesize that it could compromise the role of blood vessels during bone growth by impairing EC phenotypes. Thus, better understanding the properties of titanium-enriched medium on angiogenesis in wound healing and osseointegration process is urgently required [11, 12].

As important members within the angiogenesis process, EC is housed in the lumen of the vessel, is constantly exposed to the blood flow forces, regulates the blood supplement to tissue, and is crucial in the angiogenesis process [13]. The formation of new blood vessel supports new bone formation by transporting fundamentals constituents, such as nutrients, regulatory factors, oxygen, and osteoprogenitor cells [12]. Some authors have already reported coupling angiogenesis with osteogenesis, which are both important and dynamic processes to drive bone growth and remodeling during wound healing [14].

In bone development and healing, the angiogenesis and osteogenesis are coupled processes, through complex intercellular signaling, that also exists in the osseointegration and biomedical devices, to ensure an appositional bone development on the implanted device [15]. First, some growth factors are released by extravasate platelets from injured vessels on the alveolar bone site, such as PDGF (platelet-derived growth factor), TGF-β (transforming growth factor beta), FGF (fibroblast growth factor), and VEGF (vascular endothelial growth factor). Among them, VEGF is one of the main factors responsible for angiogenesis promotion and is crucial to osteogenesis [16].

Considering the pivotal role of ECs in wound healing [17], their biological responses to different biomaterials must be investigated. Thus, this study aims to evaluate the behavior of human umbilical vein endothelial cell (HUVEC) exposed to a titanium-enriched medium by exploring molecular approaches focusing on comprehending intracellular pathways involved with angiogenesis and cell survival, and proliferation signaling. The cascade of events for wound healing is complex and involves highly regulated processes, such as angiogenesis. The comprehension of these processes at the cellular and molecular level is far from complete [18]. In this context, genes such as VEGF and its receptors, VEGFR1 and VEGFR2, are relevant as they are closely related to the angiogenesis process and are required for tissue wound healing [19]. Downstream to activate receptor tyrosine kinases, protein kinase B (PKB or Akt) is a multifaceted protein and plays an important role in cell metabolism, growth, proliferation, and survival. Its activation is controlled by a multi-step process, mainly involving phosphoinositide-3-kinase (PI3K) [20–22]. Specifically, we evaluated whether the Ti-enriched medium was able to modulate EC performance. Summarizing, our data show that the Ti-enriched medium requires an upmodulation of PI3K/AKT signaling in ECs to maintain their angiogenic phenotype.

## Materials and methods

### Reagents, TiO_2_ alloys, antibodies, and primers

Two different titanium surfaces (discs) were investigated in this study, distinguished by the surface properties, as follows: Machined (Wo/DAE), and Dual Acid-Etched (W/DAE). The metallic TiO_2_-based alloys were generously donated by the S.I.N. (São Paulo, SP, Brazil). The cell culture flasks were purchased from TPP (Trasadingen, Switzerland), and the cell culture reagents from Nutricell (Campinas, SP, Brazil). Ripa buffer (R0278), Phosphatase inhibitor cocktail 2 (P5726), and bovine serum albumin (A7906) were purchased from Sigma Chemical Co. (St. Louis, MO, USA). Gotaq qPCR master mix (A6002) was purchased from PROMEGA (Madison, WI, USA). The following antibodies were purchased from Cell Signaling (Danvers, MA, USA): Akt Antibody (4691P, 60 kDa); PhosphoAkt (S473) Antibody (4060P, 60 kDa); Anti-Rabbit IgG (7074).

### Cell culture and PI3K inhibition

HUVECs were used in this study. ECs were cultivated in RPMI medium (Nutricell, Campinas, SP, Brazil) supplemented with penicillin (100 U/ml), streptomycin (100 mg/ml), and 10% fetal bovine serum (FBS), and maintained at 37 °C and 5% CO_2_. HUVECs were cultivated in culture flasks until proper amount and then were seeded in traditional culture dishes until adhesion, when the culture medium was exchanged for the different medium treatments as described below, for 72 h. To investigate the role of PI3K in the signaling behavior of ECs, we again carried out the treatment as described, but using a culture medium containing Wortmannin (5 μM), a PI3K inhibitor.

### TiO_2_-enriched medium obtaining

To prepare the TiO_2_-enriched medium, dual-acid-etching (DAE) treating surface (named W/DAE) and the machined surfaces (named Wo/DAE), the experimental alloys (*n* = 6) were incubated in cell culture media (RPMI) without FBS up to 24 h at 37 °C, 5% CO_2_, and 95% humidity [0.2 g/mL (w/v); ISO 10993:2016]. The TiO_2_-enriched medium contains molecules released from those metallic alloys and might affect the biology of ECs. To test this hypothesis, the TiO_2_-enriched medium was further used to treat the ECs.

### Total mRNA isolation and RT‐qPCR analysis

After treatment, the cells were harvested and total mRNA isolated using Ambion TRIzol Reagent (Life Sciences, Thermo Fisher Scientific Inc., Waltham, MA, USA) and treated with DNase I (Invitrogen, Carlsbad, CA, USA). Complementary DNA (cDNA) synthesis was performed with High Capacity cDNA Reverse Transcription Kit (Applied Biosystems, Foster City, CA, USA) according to the manufacturer’s instructions. Real‐Time qPCR was carried out in 10 μl, containing PowerUpTM SYBRTM Green Master Mix 2× (5 μl; Applied Biosystems, Foster City, CA, USA), 0.4 μM of each primer, 50 ng of cDNA, and nuclease-free H_2_O. Results were expressed as relative amounts of the transcripts using glyceraldehyde 3‐phosphate dehydrogenase (GAPDH) and 18S as reference genes (housekeeping gene), using the cycle threshold (Ct) method. Primers and run details are described in Table 1.

**Table 1:** Expression primers sequences and qPCR cycle conditions

Gene	Primer	5′-3′ Sequence	Work condition
AKT	Forward 1	CAGCGCGGCCCGAAGGAC	95 °C—3 s; 55 °C—8 s; 72 °C—20 s
	Forward 2	GGACTCCCGTTTGCGCCAGT	
	Reverse	GACGCTCACGCGCTCCTCTC	
VEGFR1	Forward	CAGGCCCAGTTTCTGCCATT	95 °C—3 s; 55 °C—8 s; 72 °C—20 s
	Reverse	TTCCAGCTCAGCGTGGTCGTA	
VEGFR2	Forward	CCAGCAAAAGCAGGGAGTCTGT	95 °C—3 s; 55 °C—8 s; 72 °C—20 s
	Reverse	TGTCTGTGTCATCGGAGTGATATCC	
VEGF	Forward	TGCAGATTATGCGGATCAAACC	95 °C—3 s; 55 °C—8 s; 72 °C—20 s
	Reverse	TGCATTCACATTTGTTGTGCTGTAG	
INOS	Forward	TGGATGCAACCCCATTGTC	95 °C—3 s; 55 °C—8 s; 72 °C—20 s
	Reverse	CCCGCTGCCCCAGTTT	
ENOS	Forward	TATTTGATGCTCGGGACTGC	95 °C—3 s; 55 °C—8 s; 72 °C—20 s
	Reverse	AAGATTGCCTCGGTTTGTTG	
GAPDH	Forward	AGGCCGGTGCTGAGTATGTC	95 °C—3 s; 55 °C—8 s; 72 °C—20 s
	Reverse	TGCCTGCTTC ACCACCTTCT	
18S	Forward	CGGACAGGATTGACAGATTGATAGC	95 °C—3 s; 55 °C—8 s; 72 °C—20 s
	Reverse	TGCCAGAGTCTCGTTCGTTATCG	

### Western blotting

After 72 h of treatment with TiO_2_-enriched medium, the challenged HUVECs were washed in ice-cold PBS and protein extracts were obtained using a RIPA lysis buffer (Sigma Aldrich, St. Louis, Missouri, USA) and supplemented with a cocktail of antiproteases and anti-phosphatases (Sigma Aldrich, St. Louis, MO, USA) up to 1 h on ice. Protein extracts were cleared by centrifugation 14,000 rpm for 15 min at 4 °C. The precipitate was then resuspended in 100 μL of RIPA lysis buffer (Sigma Aldrich, St. Louis, Missouri, USA). The protein extracts were clarified and the protein concentration determined by the Lowry method [23]. Protein extracts were resolved by SDS-PAGE and later transferred to PVDF membranes (Bio-Rad, Hercules, CA, USA). An equal volume of gel loading buffer [100 mmol L^−1^ Tris–HCl (pH 6.8), 200 mmol L^−1^ dithiothreitol, 4% SDS, 0.1% bromophenol blue, and 20% glycerol] was added to the samples and boiled for 5 min at 95 °C. Aliquots of the samples (75–100 μg/lane) were resolved into SDS-PAGE (8, 10, or 12% gels) and later transferred to PVDF membranes (Millipore, USA), which were blocked with 5% nonfat dry milk dissolved in tris-buffered saline (TBS)-Tween-20 (0.05%) and then incubated overnight with the appropriate primary antibody (1:1000) at 4 °C. After 1×-washing in TBS–Tween-20 (0.05%) and 2×-washing in TBS, the membranes were incubated with horseradish peroxidase-conjugated secondary anti-rabbit or anti-mouse IgGs antibodies (1:2000), diluted in blocking buffer for 1 h. Immunoreactive bands were detected using Enhance Chemiluminescence (ECL, Pierce, USA).

### Statistical analyses

Results were represented as mean ± standard deviation (SD). The samples assumed a normal distribution with *p* < 0.05 considered statistically significant and *p* < 0.001 considered highly significant. In the experiment with >2 groups, we used one-way ANOVA with multiple comparisons, to compare all pairs of groups. In this case, the significance level was considered when alpha = 0.05 (95% confidence interval). The software used was GraphPad Prism 7 (GraphPad Software, USA).

## Results and discussion

**Fig. 1:**
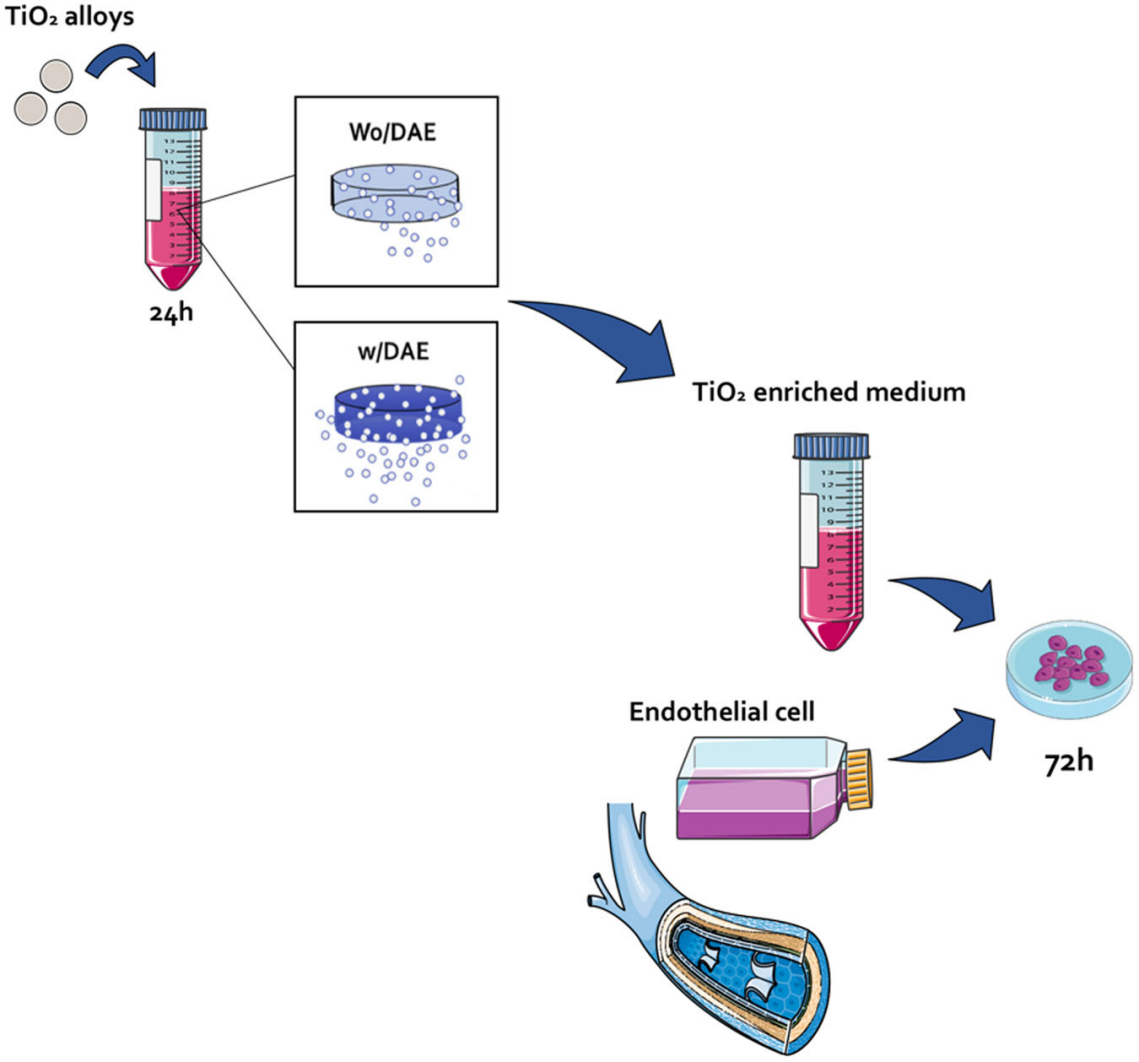
Experimental design of this study. To evaluate the biological response of endothelial cells to TiO_2_, the Ti-enriched medium was obtained by incubating Ti-based materials in the cell culture medium (FBS free), as recommended by ISO 10993:2016 (part 5). Thereafter, the Ti-enriched medium was used to challenge endothelial cells up to 72 h, when the samples were collected for the analysis. The main focus here was to evaluate whether there is some association of survival signaling an angiogenic stimulus of titanium, which could better support comprehension about their biocompatibility and dynamic relationship with the surrounding tissue

The biological responses of tissue surrounding the implanted metallic biomaterials require a well-coordinated cascade of events involving a coupled network between undifferentiated cells and angiocrine mediators released by ECs. It is known that the correct coupling between endothelium and bone guarantees proper cell differentiation and tissue regeneration, resulting in successful osseointegration. Although endothelium is not properly in close contact with the implanted materials, we have recently reported that titanium-based devices release a considerable amount of titanium and this interferes in cell metabolism [9, 10, 24–26], which could affect cells away. In this study, to evaluate whether the surface particles released from Ti affect the metabolism of ECs, we previously prepared a Ti-enriched medium and used it to further challenge HUVEC cells up to 72 h (Fig. 1). We suggest Ti-enriched medium mimics the Ti released when the devices are implanted in a host, covering an indirect and alternative model to study the effect of Ti on eukaryotic and adherent cells, as has been suggested previously. The performance of ECs was estimated by evaluating widely accepted angiogenic biomarkers at the mRNA levels, such as: VEGFR1, VEGFR2, VEGF, eNOS, and iNOS.

Within the repertoire of cascading events involved in the biological response to biomaterials, it is expected that viable cells interacting with biomaterials surfaces drive their biocompatibility [27, 28]. The surface modifications, such as DAE of titanium implants, have produced a positive effect on osseointegration in newly formed and native bone [29], triggering different signals in osteoblast-like cells [10], and our data show that these surfaces also trigger distinct responses in ECs. Experimentally, we proposed intracellular signaling pathways to evaluate cell viability mainly by considering PI3K/PKB/Akt upstream stimulus culminating in gene expression related to cell proliferation and survival [30, 31]. In addition, as these protein activities are regulated by phosphorylation, the effectivity of this signaling can be measured by the specific phosphorylation profile [32]. In turn, Akt is a serine/threonine protein kinase that is activated by a number of growth factors and cytokines in an upstream phosphatidylinositol-3 kinase-dependent manner.

**Fig. 2:**
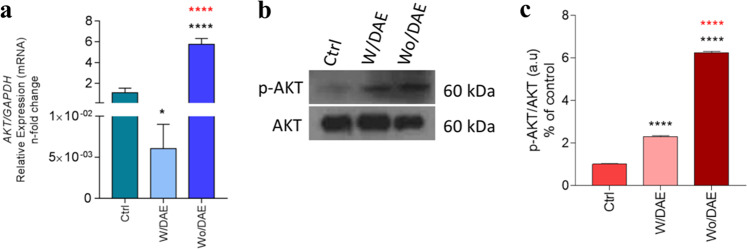
Survival signaling was evaluated by measuring the phosphorylation profile of AKT. To understand whether survival signaling was required by endothelial cells responding to Ti-enriched medium (72 h), the cells were subjected to Ti-enriched medium obtained by incubating two different conditions of titanium discs [double acid-etching (DAE) treatment (W/DAE) and without DAE (Wo/DAE)] into cell culture medium up to 24 h. After subjecting the cells up to 72 h, the samples were obtained to allow the gene expression of AKT (**a**) and protein performance (**b**, **c**) by evaluating AKT phosphorylation. GADPH was considered a housekeeping gene. Statistics: the value obtained to Ctrl was considered 1, and the relative values obtained to W/DAE or Wo/DAE are shown in fold‐changes. One-way ANOVA with multiple comparisons were applied to compare all pairs of groups. Differences were considered significant when *p = 0.04 and ****p < 0.0001 when compared with the Ctrl; and ****p < 0.0001 when compared with W/DAE group

Here, the PI3K/Akt signaling was evaluated by measuring the phosphorylation profile of Akt at S473. Phosphorylation of Akt at S473 guarantees its activity, leading to additional substrate-specific phosphorylation events in both the cytoplasm and nucleus, including inhibitory phosphorylation of the pro-apoptotic FOXO proteins [33, 34]. Figure 2 shows that the phosphorylation of PKB/Akt was significantly increased in response to Ti-enriched medium, in both evaluated surfaces: w/DAE and wo/DAE. Importantly, our data demonstrated an upexpression of mRNA of Akt in ECs responding to the Ti-WoDAE-enriched medium (Fig. 2a). Variations in titanium surfaces can modulate the PI3K/Akt pathway differently in osteoblast-like cells [35], and we observed that distinct titanium surfaces developed different modulations in this pathway in ECs, seen in the changes to the gene expression and protein AKT phosphorylation pattern. Altogether, these data strongly suggest the importance of this signaling in challenged ECs, maybe modulating their viability and phenotype, as fully active PKB/Akt mediates numerous cellular functions including angiogenesis, metabolism, growth, proliferation, survival, protein synthesis, and transcription [33, 36].

**Fig. 3:**
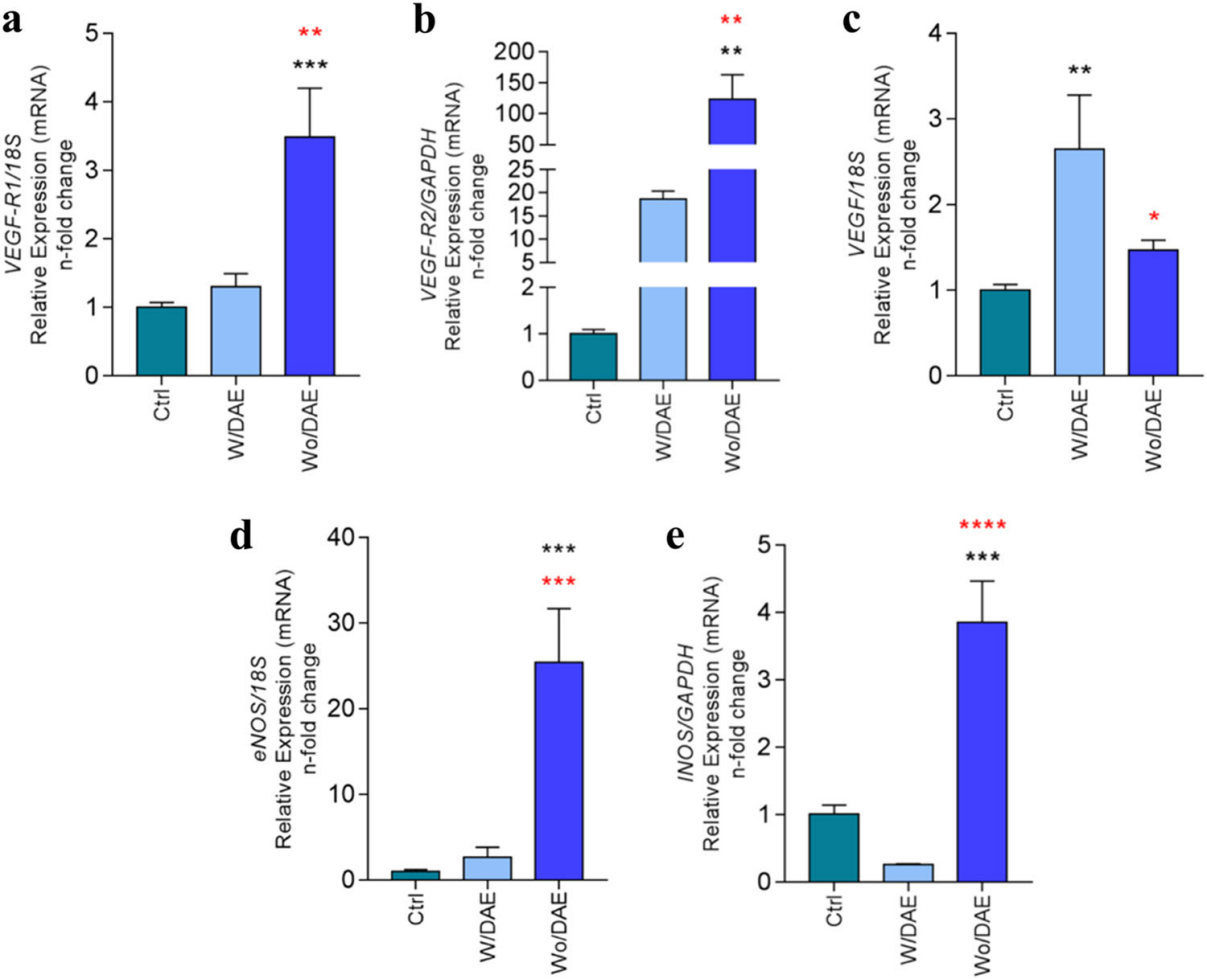
Angiogenic stimulus of titanium (Ti) on endothelial cells. The samples were collected respecting the already described methodology to allow qPCR technology performance. To address the angiocrine effect of Ti on endothelial cells, we investigated VEGFR1 (**a**), VEGFR2 (**b**), VEGF (**c**), eNOS (**d**), and iNOS (**e**) genes. The GADPH gene was considered the housekeeping gene and used to normalize the values. Statistics: One-way ANOVA with multiple comparisons was used to compare all pairs of groups, and the differences were considered significant when **a** VEGFR1 ***p = 0.0009 and **p = 0.0019; **b** VEGFR2 **p = 0.0014 and **p = 0.0032; **c** VEGF **p = 0.004 and *p = 0.02; **d** eNOS ***p = 0.0006 and ***p = 0.0008; and **e** INOS ***p = 0.0002 and ****p < 0.0001. Black (*) when compared with Ctrl and red (*) when compared with W/DAE

Later, the repertoire of genes involved with angiogenic phenotype was also investigated, and Fig. 3 provides a panorama where Ti medium enriched by Wo/DAE condition requires a significant activity of those genes, such as VEGFR1 (Fig. 3a), VEGFR2 (Fig. 3b), VEGF (Fig. 3c), iNOS (Fig. 3d), and eNOS (Fig. 3e). Importantly, VEGFR2 (Fig. 3b) and VEGF (Fig. 3c) genes are stimulated by both surfaces investigated here (W/DAE and Wo/DA). This different biological consequences between the surfaces can be explained by the concentration of titanium released into the medium. To date, VEGF has long been recognized as the key regulator of vascular development and function in health [37]. In addition, Kitamura et al. [38] demonstrated that the Akt/Girdin signaling pathway is essential in VEGF-mediated postneonatal angiogenesis. Nonetheless, they reveal that Girdin knockdown severely impairs cell migration and tube formation by HUVECs, and that Girdin^−/−^ mice display significant defects in postnatal microvascular remodeling in the retina and the sprouting of vessels from aortae [38]. In conjunction, it seems acceptable to suggest that Ti released by implantable devices could also dynamically modulate the phenotype of ECs, which explains, in association with their effect on osteoblasts [9, 10, 25, 26, 39–42], the success of titanium in implantology applications, since angiogenic factors by ECs are required to drive angiogenesis that modulates the traffic of nutrients, growth factors, and undifferentiated cells to the site where the tissue is regenerate. We are convinced there is a coupling of mechanisms considering that cells respond to titanium directly (mainly osteogenic cells adhering on the surfaces directly) and indirectly (undifferentiated cells and ECs/angiogenesis), where these devices coordinate the surrounding microenvironment and drive the osseointegration-related mechanism.

**Fig. 4:**
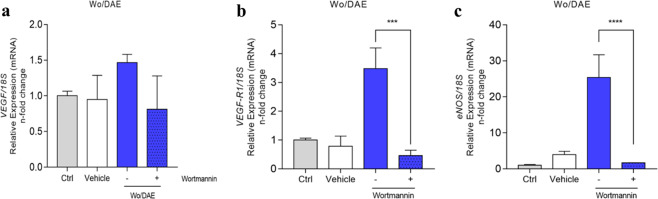
Wortmannin targeting PI3K suppresses the angiogenic stimulus of titanium (Ti). The samples were collected respecting the already described methodology to allow qPCR technology performance. To address whether PI3K/AKT is involved in endothelial responding to the Ti-enriched medium, we reanalyzed the already known performance of genes, as was shown in Fig. 3, but now treated with wortmannin, a specific chemical inhibitor of PI3K (upstream to AKT). Thus, the suppression of the angiogenic stimulus by Ti is clear since VEGF (**a**), VEGFR1 (**b**), and eNOS (**c**) genes were significantly down expressed when PI3K was inhibited. The 18S gene was considered the housekeeping gene and used to normalize the values. Statistics: The statistical analysis test used was one-way ANOVA with multiple comparisons to compare all pairs of groups, and the figure specifies the differences between the absence (−) and the presence (+) of the Wortmannin. Differences were considered significant when **b** VEGFR1 ***p = 0.0002 and **c** eNOS ****p < 0.0001

We noticed a potential involvement of survival signaling in modulating angiogenic stimulus in response to the Ti-enriched medium since there was a probable correlation between PKB/AKT performance and genes related to angiogenic signals, mainly in the Wo/DAE group (see Figs. 2 and 3). Finally, to test the hypothesis that PI3K/AKT drives angiogenic factors in response to the Ti-enriched medium, we subjected the cells to a chemical inhibition of PI3K using well-known Wortmannin, as it is widely proposed by others [43], and further investigated the behavior of VEGF (Fig. 4a), VEGFR1 (Fig. 4b), and eNOS (Fig. 4c) genes. Our data clearly shows that wortmannin suppresses the angiogenic factors because all of these three genes were significantly downregulated (Fig. 4). Altogether these data validate our hypothesis that PI3K/Akt signaling is involved in angiogenic signals triggered by tienriched medium. Mechanistically, VEGF induces nitric oxide production and release by nitric oxide synthase (NOS) in isolated vessels and in cultured ECs [44–46], which is attenuated by PI3K inhibitors [47]. Subsequently, it was demonstrated that VEGF stimulates PKB/Akt-mediated eNOS phosphorylation at Ser1177 [36, 48, 49].

Thus, we reasonably suggest that PI3K/AKT signaling participates in the angiogenic phenotype of ECs responding to the Ti-enriched medium. We previously reported that the major mechanotransduction pathway activates PI3K, which plays a pivotal role in guaranteeing EC phenotype and vascular homeostasis [43], and Ti-release displays a very similar effect, mainly in response to Wo/DAE.

Peri-implant healing seems to recapitulate conventional regenerative processes involving a coupling of biological systems, which requires a cascade of carefully and precisely regulated steps and events that correlate with the appearance of various cell types during distinct stages of healing [50–54]. Although titanium has been considered a physiologically inert material, we have demonstrated Ti develops dynamic interaction with the microenvironment by releasing particles and active molecules affecting osteoblast biology by positively coordinating proliferative and differentiation mechanisms, and the properties of the surfaces seems to influence this response [16]. These modifications on titanium surfaces aim to optimize the wound healing to reduce the clinical time for patient recovering and has been shown to trigger changes in osteoblast metabolism [24]. A previously published study indicated involvement of a set of genes related with osteogenesis in response to dual-acid-etched surface of titanium in human mesenchymal stromal cells [55]. The involvement of ECs has a pivotal role during crucial phases of wound healing. Their importance on osseointegration success has raised some questions mainly about the response to changes in titanium surfaces [6], and our data address this issue and provides further comprehension.

This study reinforces that the titanium-released particles modify differentially the EC behavior emphasizing the importance of survival and angiogenic related intracellular signaling in these responses by upactivating PI3K/AKT signaling, mainly in response to Wo/DAE. In conjunction, these data support that titanium-implantable devices interact dynamically with the host surrounding tissues modulating the activity of osteogenic and ECs, maybe coupling osteogenesis and angiogenesis processes in peri-implant tissue and then contributing with their successful osseointegration.
